# Nesprin-1: novel regulator of striated muscle nuclear positioning and mechanotransduction

**DOI:** 10.1042/BST20221541

**Published:** 2023-05-12

**Authors:** Shanelle De Silva, Zhijuan Fan, Baoqiang Kang, Catherine M. Shanahan, Qiuping Zhang

**Affiliations:** 1 King's College London British Heart Foundation Centre of Research Excellence, School of Cardiovascular and Metabolic Medicine & Sciences, London SE5 9NU, U.K.; 2 Clinical Laboratory, Tianjin Third Central Hospital, Tianjin 300170, China

**Keywords:** DCM and EDMD, mechanotransduction, microtubule, nesprin, nuclear envelope LINC complex, nuclear positioning

## Abstract

Nesprins (nuclear envelope spectrin repeat proteins) are multi-isomeric scaffolding proteins. Giant nesprin-1 and -2 localise to the outer nuclear membrane, interact with SUN (Sad1p/UNC-84) domain-containing proteins at the inner nuclear membrane to form the LInker of Nucleoskeleton and Cytoskeleton (LINC) complex, which, in association with lamin A/C and emerin, mechanically couples the nucleus to the cytoskeleton. Despite ubiquitous expression of nesprin giant isoforms, pathogenic mutations in nesprin-1 and -2 are associated with tissue-specific disorders, particularly related to striated muscle such as dilated cardiomyopathy and Emery–Dreifuss muscular dystrophy. Recent evidence suggests this muscle-specificity might be attributable in part, to the small muscle specific isoform, nesprin-1α2, which has a novel role in striated muscle function. Our current understanding of muscle-specific functions of nesprin-1 and its isoforms will be summarised in this review to provide insight into potential pathological mechanisms of nesprin-related muscle disease and may inform potential targets of therapeutic modulation.

## Introduction

The nuclear envelope (NE) is a highly dynamic organelle providing spatial separation of the nuclear and cytoplasmic environments and protecting the genome encapsulated inside. The NE is formed of two bi-lipid membranes, an inner and outer nuclear membrane (INM and ONM), separated by the perinuclear space (PNS) and perforated by nuclear pore complexes (NPCs) that regulate the transport of macromolecules between the nucleoplasm and cytoplasm [1]. The structure and function of the NE is supported by the LInker of Nucleoskeleton and Cytoskeleton (LINC) complex. This complex bridges the nucleus to the three major cytoskeletal elements (actin filament-F-actin, microtubule-MT and intermediate filament-IF) and is necessary to maintain nuclear homeostasis by providing essential mechanical connections throughout the cell, in order to maintain nuclear integrity and morphology, as well as regulate nuclear positioning, mechanical signalling and gene expression [2].

**Figure 1.**
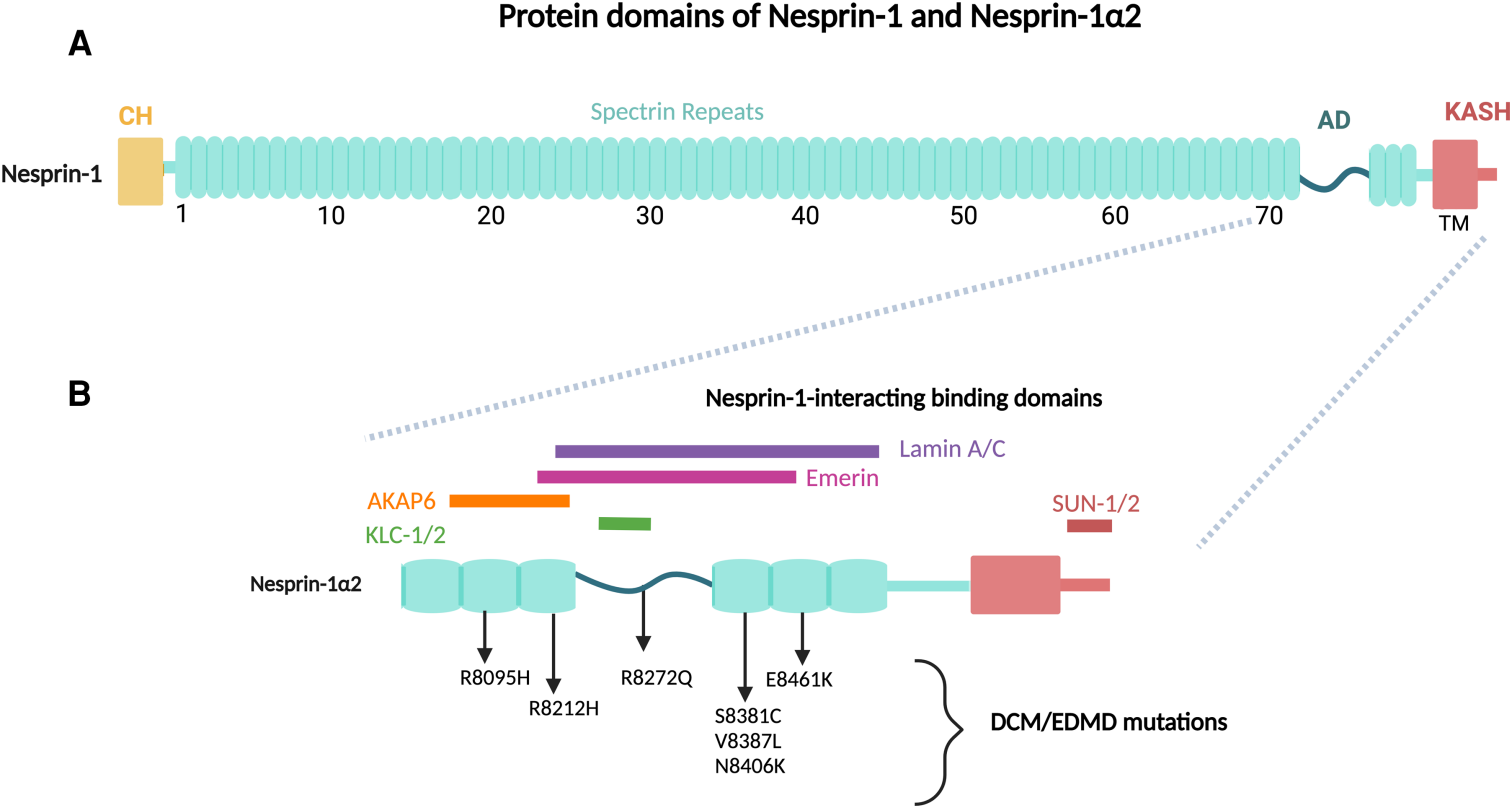
Protein domains of nesprin-1G and nesprin-1α2. (**A**) Giant nesprin-1 structure consists of three major domains: (i) tandem actin binding CH domains (yellow) (ii) central rod of SRs (blue) (iii) NE-targeting KASH domain, consisting of a transmembrane (TM) domain and luminal KASH peptide (red). Additionally, a highly conserved-terminal adaptive domain (AD) at C-terminus serves to structurally stabilises the spectrin repeat (SR)s. (**B**) Nesprin-1α2 is a small muscle specific isoform, equivalent to the C-terminal region of nesprin-1G containing the last six spectrin repeats and the KASH domain. Nesprin-1 interacting protein binding domains and validated DCM/EDMD pathological mutations are indicated within this highly conserved C-terminal region.

**Figure 2.**
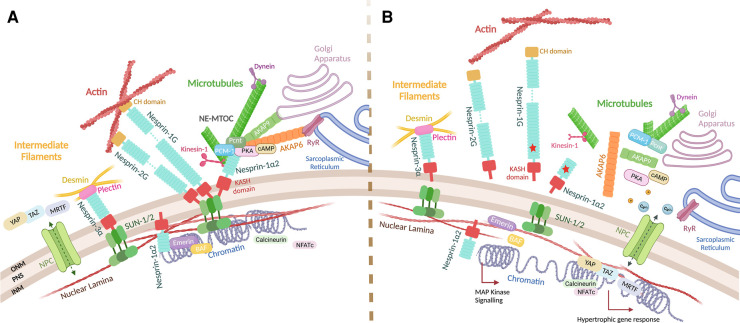
Roles of the nesprins and the LINC complex at the nuclear envelope. (**A**) Nesprin isoforms localise at the ONM interact with SUN1/2 to form the LINC complex, physically linking the cytoskeleton to the nucleus. The LINC complex associates with the chromatin through interactions with the nuclear lamina and chromatin binding partners such as emerin and BAF. At the ONM nuclear membrane, giant nesprin isoforms-1 and -2 can directly bridge the nucleus to the actin cytoskeleton, whereas nesprin-3 associates with the muscle-specific intermediate filament protein desmin via plectin, and short nesprin isoform nesprin-1α2 associates with MTs via KLC-1/2 and AKAP6. The scaffolding protein AKAP6 can in turn anchor centrosomal proteins, PCM1, PCNT, AKAP9 to the NE, and form a cAMP-PKA signalling hub, which may have a role in NE calcium handling via interacting with RyRs at the sarcoplasmic reticulum. Smaller nesprin isoforms can also be found at the INM where they interact with lamin A/C and emerin. (**B**) Nesprin mutations (red stars) may cause LINC complex disruption and uncoupling of the cytoskeleton from the nucleus, resulting in structural defects such as abnormal nucleus shape, size, migration or positioning, or pathophysiological activation of signalling pathways or mechanosensitive transcription factors, which ultimately leading to cellular dysfunction and cardiac and skeletal muscle myopathies.

One of the major components of the LINC complex is the evolutionarily conserved multi-isomeric scaffolding family of nesprin (Nuclear Envelope Spectrin Repeat) proteins. To date, six mammalian genes: *synaptic nuclear envelope (SYNE)-1, -2, -3, -4, KASH5 and LRMP* have been identified, encoding for Klarsicht/ANC-1/Syne Homology (KASH) domain-containing proteins: nesprin-1, -2, -3, -4, KASH5 and lymphoid-restricted membrane protein, respectively [3–7]. Full length nesprin-1 and -2, known as ‘giant’ isoforms, are the second and third largest known proteins with molecular masses of 1.01 MDa (146 exons) and 796 KDa (116 exons) respectively, sharing 64% homology. These giant proteins were originally identified in screens for novel vascular smooth muscle cell differentiation markers and synaptic muscle components, but have since been shown to be ubiquitously expressed [5–9]. Giant nesprin-1 and -2 contain three major domains: an N-terminal paired Calponin Homology (CH) domains that interact with the actin cytoskeleton, a C-terminal Klarsicht/ANC-1/Syne Homology (KASH) domain that is targeted to the NE, and a central rod domain-containing multiple spectrin repeats (SRs) connecting the CH and KASH domains and mediating protein–protein interactions (Figure 1A) [10, 11]. Of note, there is a highly conserved adaptive domain (AD) at the C-terminus of nesprin-1 and -2, that acts to structurally stabilise the SRs in nesprin molecules [12, 13]. Giant nesprin-1 and -2 are targeted to the ONM by the KASH domains, which interact with a homo-trimer of Sad1p/UNC-84 (SUN) domain-containing proteins, either SUN1 and/or SUN2, via the SUN domains in a heterohexameric assembly in the PNS, which forms the core of the LINC complex [14–16] (Figure 2A). The LINC complex is formed by further association with the NE protein emerin, and the nuclear lamina that underlies the NE, and consists of a network of the intermediate filament proteins lamin A/C, lamin B1 and lamin B2 [17, 18]. The nuclear lamina is closely associated with the chromatin at the nuclear periphery through anchorage of heterochromatin, producing a continuous route for force transmission from the cytoskeleton to the genome and a mechanism for direct gene regulation via the LINC complex [1, 19].

The importance of the LINC complex in muscle physiology is evidenced by the large number of mutations in its components causing a family of diseases known as nuclear envelopathies, which include a wide range striated muscle disorders, i.e affecting cardiac and skeletal muscle, such as dilated cardiomyopathy (DCM) and Emery–Dreifuss muscular dystrophy (EDMD). Striated muscles have a highly specialised organisation of sarcomeres, which actively generate contractile forces throughout life, resulting in an unique mechanical environment of the nucleus; an important consideration in investigating how ubiquitously expressed LINC complex components can disproportionately affect muscle tissues. These mutations identified in DCM and EDMD are suggested to disrupt the LINC complex and cause nucleocytoskeletal uncoupling resulting in structural defects and chromatin disorganisation, that manifest as defects in nuclear morphology, myonuclear positioning and distribution, aberrant mechanotransduction and gene regulation, leading to pathogenesis of muscle disorders [11, 20–23].

Numerous isoforms of nesprin-1 and -2 are produced by alternative initiation/termination of transcription and/or alternative splicing of the giant *SYNE1* and *SYNE2* genes, which may differentially contribute to cellular functions. These shorter isoforms, which lack either the CH domains, KASH domain, or have a varying number of SRs, show tissue-dependent expression and exhibit different subcellular locations. For example, some small nesprin isoforms, e.g. nesprin-1α2, localise at both ONM and INM, while other nesprin-1 and -2 isoforms have been shown to localise at the sarcomeric Z-line of both human skeletal and cardiac muscle as well as at focal adhesions, the Golgi apparatus, and actin stress fibres across multiple cell types [24–27]. Of note, three small isoforms, nesprin-1α2, nesprin-2α1 and nesprin-2ε2, were identified to be highly and almost exclusively expressed in cardiac and skeletal muscle. These isoforms are composed of evolutionarily conserved regions of nesprin-1 and -2 corresponding to the C-terminal SRs and the adjacent unstructured AD domain, which contain the binding motifs for several known interactors of nesprin-1 and -2, including lamin A/C and emerin [12, 28]. The small nesprin-1 isoform, nesprin-1α2, is a 112 kDa isoform equivalent to the last six SRs of the C- terminal region of nesprin-1 giant (Figure 1B), generated by an alternative start site and including a unique 31 amino acid N-terminal region [12, 24]. Nesprin-1α2 is dramatically up-regulated during muscle differentiation, where it localises to the ONM and associates with the MT network by highly conserved binding domains for kinesin light chain-1/2 (KLC-1/2), a subunit of the tetrameric MT motor protein kinesin-1, and one of the centrosomal proteins, alpha kinase anchoring protein 6 (AKAP6), a key component of the NE MT-organising centre (MTOC) (Figure 1B), suggesting a novel role for nesprin-1α2 in striated muscle function via perinuclear MT interactions [22, 29–34]. Importantly, pathogenic mutations of striated muscle disorders tend to cluster within the C-terminal region of nesprin that correspond to nesprin-1α2, indicating functional importance of this region to striated muscle physiology (Figure 1B) [35].

This review will explore the role of nesprin-1 and its interactions with cytoskeletal proteins that facilitate the functioning of cardiac and skeletal muscle, and their common features such as formation of the NE-MTOC and mechanosignalling pathways. However, it is important to consider the broad family of homologous nesprin isoforms, in particular research relating to highly homologous nesprin-2 C-terminal may provide some insight into overlapping functions. Moreover, consolidation of recent literature suggests a novel role for nesprin-1α2 through its interactions with MTs in muscle function in addition to, but not mutually exclusive of, the previously described functions of the LINC complex. Although the mechanics of myonuclei are vastly more complex, this review will provide a reductionist understanding of the contribution of nesprin-1, and putative role of nesprin-1α2, to muscle and provide novel insights into how its associations at the NE may mediate striated muscle defects in NE-related pathophysiology**.**

## Roles of nesprin-1/1α2 and the LINC complex in striated muscle

### Microtubule and nuclear organisation of striated muscle

The cytoplasmic extension of nesprin isoforms allows direct and indirect interactions with the three major types of cytoskeletal proteins: F-actin, MTs, and IFs (Figure 2A). Coordinated action of the cytoskeletal networks is crucial for myonuclei movement, positioning, force transmission and contractile function, therefore, disruption of any component or compromised integrity of the LINC complex can result in cellular dysfunction and ultimately to cardiac or skeletal myopathies [36–38] (Figure 2B). To understand the pathological mechanisms underlying this phenomenon, it is important to establish the key structural elements of striated muscle to which nesprins tether myonuclei. Cardiac and skeletal muscle are collectively known as striated muscle due to the arrangement of thick and thin filaments arranged into a repeated contractile apparatus known as sarcomeres. Whilst sarcomeric actin functions with myosin to produce contractile force, F-actin, MTs and IFs exist as a highly integrated and dynamic support network tethered to myonuclei via the LINC complex. This network extends throughout the cell to the plasma membrane [35, 39, 40], where cytoskeletal filaments can interact with specialised complexes such as costameres, focal adhesions or intercalated discs (IDs) in cardiac muscle, which facilitate intracellular signalling and transmission of extracellular cues to the nucleus [41, 42].

**Figure 3.**
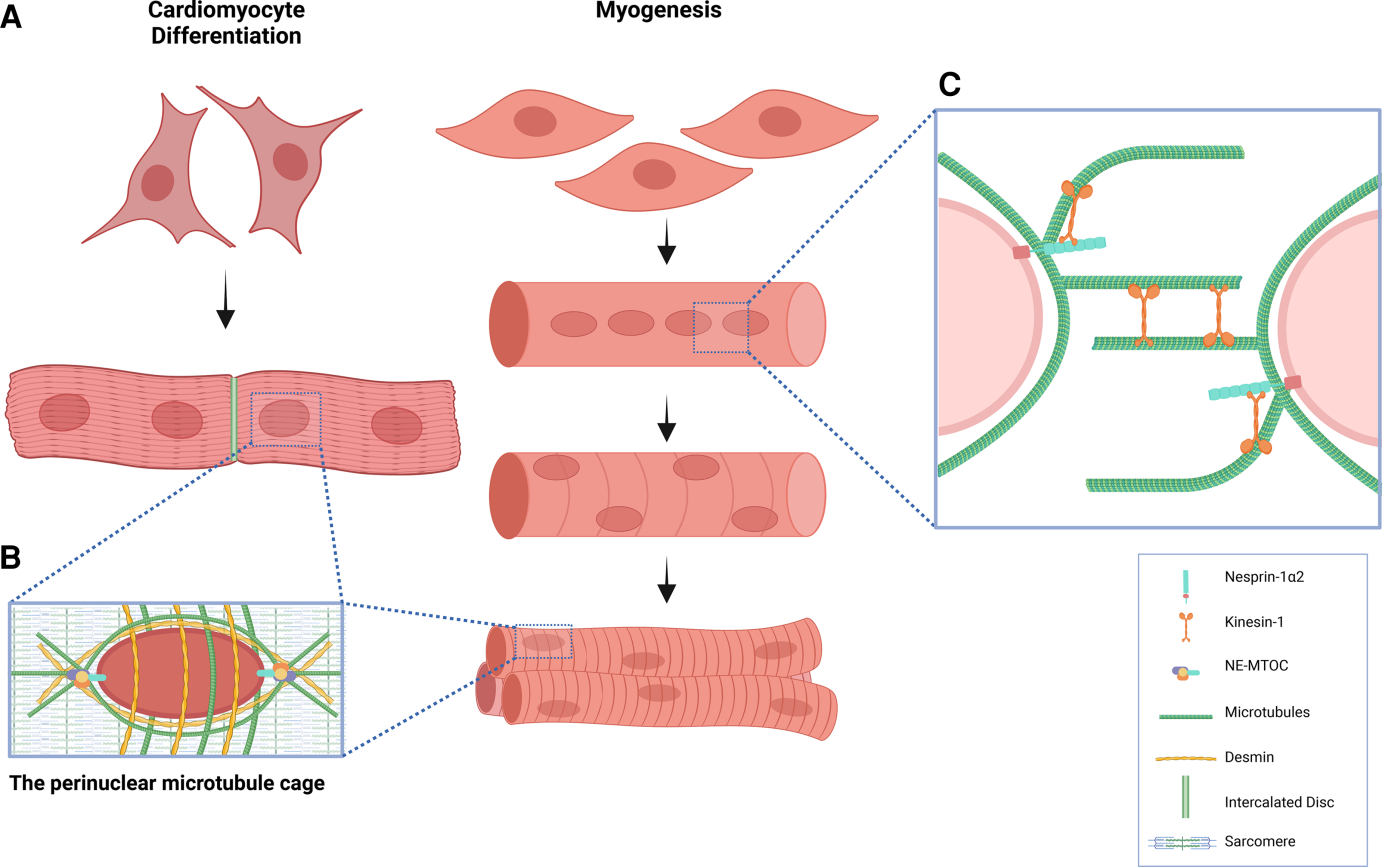
Microtubules in nuclear positioning and organisation in striated muscle cells. (**A**) Cardiomyocyte differentiation results in mono- or bi-nucleated cells, where the nuclei are centrally positioned, and cells are extensively interconnected by IDs. Conversely, during myogenesis myoblasts fuse into a multi-nucleated myofibers, where nuclei are peripherally anchored amongst sarcomeres. (**B**) In both skeletal and cardiac muscle cells, MT nucleation can occur from the nucleus as centrosomal proteins are recruited by nesprin-1α2 via AKAP6 to the NE facilitating the formation of the perinuclear MT cage. (**C**) During myogenesis, the association of nesprin-1α2 with MTs, via the MT motor kinesin-1, mediates the dynamic movement of nuclei by either transforming the nucleus into a kinesin-1 cargo on MT tracks, or by exerting repelling forces through cross-linked MTs of neighbouring nuclei until equidistant.

Myonuclei positioning and organisation is vastly different between skeletal and cardiac muscle cells (Figure 3A). Skeletal muscle is formed of multinucleated myotubes from the iterative fusion and differentiation of specialised precursor cells known as myoblasts. Multinucleated myotubes mature into myofibers with an ordered arrangement of peripherally anchored nuclei amongst a regular assembly of sarcomeres [43, 44]. Conversely, cardiomyocytes are mononucleated cells, or often binucleated in rodents, with a centrally localised nucleus. Cardiomyocytes do not fuse but form a functional syncytium via extensive intracellular coupling through IDs enabling synchronised contraction [45, 46]. Though nuclei arrangement differs, cardiac and skeletal muscle share commonalties in the structural adaptations of the cytoskeletal network that generate and withstand constant mechanical force.

One such feature is the shift of MT nucleation from the centrosome to the NE during terminal differentiation, enabling the formation of a perinuclear cage which spans into an interfibrillar MT network distributed between the myofilament bundles in cardiomyocytes and skeletal muscle myofibers (Figure 3B). The NE-MTOC and perinuclear cage contribute to muscle function in several ways; (i) facilitate MT mediated nuclear mobility, particularly during myogenesis, (ii) modulate contractility and confer mechanical resistance during muscle contraction, (iii) protects genome integrity, and (iv) mediates mechanotransduction by integration with the contractile apparatus and LINC complex at the nucleus. [33 47–50].

### The nuclear envelope MT organising centre

Proliferating cardiac and skeletal muscle precursor cells possess a centrosomal MTOC that nucleates MTs to form spindle fibres in order to accomplish chromosome segregation during mitosis. A hallmark of terminal differentiation is the attenuation and redistribution of the centrosomal MTOC components to various subcellular locations [51]. Unique to striated muscle and neurons, centrosomal proteins are recruited to the NE by association with AKAP6, which together with AKAP9, are anchored by nesprin-1α2 to form an NE-MTOC [52, 53]. Centrosomal proteins and MT nucleation are additionally observed at the Golgi apparatus which is uniquely distributed around the nucleus of striated muscle cells, yet the integration and contribution NE-MTOC activity at the Golgi is poorly understood [54–56].

Formation of a NE-MTOC occurs concomitantly with the differential expression and up-regulation of nesprin-1α2 [30, 33], with the N-terminal domain of nesprin-1α2 (equivalent to nesprin-1 giant SR 72 and 73) directly interacting with AKAP6β (referred to as AKAP6), a muscle-specific scaffolding protein [34, 57]. Recent studies suggest, upon myogenic differentiation, up-regulation of myogenic regulatory factors (MRFs) promote the expression of nesprin-1α2 and AKAP6 as an initial trigger for NE-MTOC formation, creating a scaffolding platform for the NE targeting of centrosomal proteins AKAP9, pericentriolar material-1 (PCM-1), and pericentrin (PCNT), which form the MTOC at the NE [50, 58]. Moreover, a BioID proximity study revealed nesprin-1α2 to be closely associated with AKAP6, AKAP9 (also known as AKAP450), PCM-1, and PCNT in myotubes (Table 1) [33]. Furthermore, depletion of nesprin-1 resulted in the loss of PCM-1, PCNT and γ-tubulin from myotube nuclei [50]. Patient derived myoblasts lacking nesprin-1α2 by a *SYNE1* nonsense mutation (25360 G > T) exhibit mislocalisation of centrosomal proteins, together indicating a unique role for nesprin-1α2 as the NE-MTOC anchor protein in striated muscle cells [58].

**Table 1:** Nesprin-1 binding partners

	Nesprin-1 partner	Binding region	Associated with	Reference
LINC complex	Lamin A/C	AD-SR74 (467-1109aa N1α2)	SUN1/2, Chromatin, >80 NETS: emerin, LAP1/2a, LBR, MAN1. Signalling pathways: Wnt/β-catenin, TGFβ, SMAD, and MAPK	[118, 119]
	Emerin	SR71-SR73 (368–627aa N1α2)	Lamin A/C, BAF	[120]
	SUN 1/2	KASH domain	Lamin A	[10, 14]
MT/MTOC	KLC-1/2	LEWD motif within AD	Microtubules	[121]
	AKAP6/AKAP6β	SR70-71	Centrosomal proteins, PKA, RyR, calcineurin	[34]
	AKAP9	nd*	Centrosomal proteins	[33]
	PCM-1	nd	Centrosomal proteins	[50]
	EB1	nd	MSP300 -Drosophila nesprin-1 orthologue	[82]
	sk-CIP	nd	LINC complex, Centrosomal proteins	[122]
OTHERS	F-Actin	CH domain	Actin filaments	[26, 123]
	FHOD1	SR18	Actin filaments	[103]
	BIN1	nd	N-WASP	[73]
	BicD2	nd	Dynein	[65]
	FAK	nd	Integrin β, focal adhesion complex	[124]

1 nd: not yet determined.

In cardiomyocytes, AKAP6 is similarly targeted to the NE by nesprin-1α2, acting as an adaptor between nesprin-1α2 and PCNT or AKAP9 [59]. Disruption of the nesprin-1 KASH domain resulted in mislocalised MT cytoskeletal components from the NE, as well as abnormal nuclear morphology and positioning in isolated adult cardiomyocytes [60]. However, in contrast with skeletal muscle, cardiomyocytes do not express myogenin which is suggested to trigger MTOC rearrangements during myogenesis; thus, the initiation of MTOC rearrangement in cardiac cells remains to be discovered. Nesprin-1α2, AKAP6, and AKAP9 were shown to form a protein platform tethering the Golgi to the nucleus, that is required for proper induction of downstream cardiac hypertrophy cascades, suggesting cell-specific functions of perinuclear MTs [59]. Further investigation is necessary to fully understand the mechanisms involving nesprin-1α2 and AKAP6 in cardiomyocyte differentiation. Current knowledge in cardiac muscle cell differentiation and nuclear movement is limited due to technical difficulties of studying cardiomyocytes *in vitro*. Nonetheless, nesprin-1 knockdown alters differentiation of embryonic stem cells to mature cardiomyocytes, furthermore, nesprin-1α2 knockout (KO) mice show high perinatal lethality suggesting nesprin-1α2 that is indispensable for embryonic development [61, 62].

Whether relocalisation of the MTOC from the centrosome to the NE in striated muscle is a cause or effect of cell cycle exit is not known, but it is evident that the NE-MTOC promotes an advantageous reorganisation of MTs around the nucleus, permitting novel mechanisms of nuclear movement and protection against the increased hemodynamic and mechanical load in developing cardiac and skeletal muscle [37].

### Nuclear positioning and myogenesis

Following the formation of the NE-MTOC, nesprins-1 and -2 further underpin myogenesis, a process unique to skeletal muscle, by anchoring the cooperative cytoskeletal networks that mediate nuclear movement and positioning following the dynamic nuclear rearrangement during myoblast differentiation into multinucleated myofibers. Current knowledge of nuclear movement predominantly stems from *in vitro* models of myogenesis, such as differentiation of C2C12 mouse myoblast cell line, demonstrating that initial nuclear alignment and distribution during cell fusion are driven by MTs, whereas actin and IFs subsequently mediate nuclei anchorage to the cell periphery as contractile myofibers form [37, 63, 64].

For myogenesis to occur, nesprin-1α2 and PCM-1 of the NE-MTOC are required for the nuclear targeting of MT motor proteins, dynein and kinesin, which regulate myonuclear alignment and positioning [50]. MT mediated nuclear movement largely relies on the interaction between nesprin-1α2 and kinesin-1, in particular one of its subunits — KLC-1/2 [32]. Kinesin-1 is a heterotetramer of two kinesin heavy chain (KHC) subunits and two KLC-1/2 subunits, which travels and transports cargo towards the plus-ends of MTs [65]. KLC-1/2 interacts with nesprin-1 and -2 through a conserved tryptophan-acidic ‘LEWD’ motif of the AD region at the highly conserved C-terminus of giant nesprin-1 and -2 that corresponds to nesprin-1α2 [22, 32]. Interestingly, nesprin-1α2 may contain a binding site for BicD2, a regulator of cytoplasmic dynein, as nesprin-1α2 is highly homologous nesprin-2 C-terminal region that was shown to interact with BicD2 [66]. Recruitment of AKAP9 by nesprin-1α2 is essential for nuclear positioning as knock down of AKAP9, caused a significant reduction in MT nucleation from the NE and led to nuclear clustering in myotubes, independently of the nesprin-kinesin binding domain [33].

Currently, there are two non-mutually exclusive models proposed for initial nuclear movement during myogenesis, both dependent on the anchorage of MTs by nesprin-1 at the NE via KLC-1/2. The first relies on MT nucleation from the NE-MTOC, where kinesin-1 and MT Associated Protein 7 can cross-link MTs, so that they repel each other until they are equidistant, as demonstrated by *in silico* modelling [33, 67]. A second mechanism depends on the LINC complex and nesprin-1 and -2 interaction with kinesin-1, where the nucleus is transformed into a kinesin-1 cargo by interaction of the nesprin/KLC-1/2 complex, which is capable of translocating to the nucleus on the antiparallel MT network formed between the MTOC of the other numerous nuclei throughout the myotube (Figure 3C) [31, 32]. Mice harbouring a conditional KO of kinesin-1 in muscle die at birth with severe muscle dystrophy and centrally localised, aggregated nuclei [68].

Nuclear aggregation and improper positioning are also frequently observed in nesprin mutant muscle cells, this is associated with disturbance to nesprin-MT interactions. Nesprin-1α2 KO mice display aligned but aggregated nuclei from isolated embryonic myofibers due to an incapacity to recruit kinesin-1 to the NE [62]. Overexpression of nesprin missense mutations, R8272Q, S8381C, and N8406K, identified in DCM patient screening, disrupted interactions between nesprin-1 and kinesin-1 resulting in defective myogenesis, evidenced by misregulation of MRFs and irregular nuclear morphology and positioning in mouse C2C12 myoblast differentiation [22].

Although myonuclei positioning is dramatically different between skeletal and cardiac muscles i.e. peripheral vs. central, it can be theorised they both utilise common interactions at the nucleus, however precise mechanisms of nuclear positioning in cardiomyocytes are yet to be established. The effect of DCM mutations (R8272Q, S8381C, and N8406K) on skeletal muscle was unknown or potentially overlooked in patients presenting with DCM and heart failure, however that these mutations relating to a cardiac phenotype can result in defective skeletal myogenesis reinforces the potential for common mechanisms within striated muscle cells. Nuclear mispositioning has also been observed in cardiac tissue, as isolated cardiomyocytes from nesprin-1 and/or nesprin-2 KO mice show decreased internuclear distances- altered nuclear distribution could potentially be attributed to disrupted kinesin-1 interactions at the nucleus, in parallel to skeletal muscle [69]. Nuclei are ultimately anchored to the periphery of the cell, predominantly distributed along the myofiber with maximal intranuclear spacing forming optimal myonuclear domains [70, 71]. Myofibril cross-linking and contraction drives the nucleus to squeeze to the periphery, a process dependent on the intermediate filament desmin, supported by actin dynamics [71]. The C-terminus of nesprin-2 was identified to directly interact with BicD and its binding partner amphiphysin 2 (BIN1), that modulate actin dynamics and nuclear positioning through N-Wasp, an activator of ARP 2/3 (Table 1). Actin polymerisation by the Arp2/3 complex contributes to organising the desmin network necessary for myofibril cross-linking [72, 73]. Depletion of desmin or nesprin-1 results in similar defects of nuclear anchorage, a phenotype exacerbated in the double KO mouse model of desmin and nesprin-1, resulting in decreased strain transmission, more severe muscle dystrophy and increased mortality [74]. A second subset of nuclei aggregate by a separate mechanism at the myotendinous junction and neuromuscular junction, structures that mediate muscle stretching and excitation respectively, in which nesprin-1 participates as KASH truncations lead to anchoring defects of both synaptic and non-synaptic nuclei [75]. However, characterisation of nuclear anchoring is limited by the lack of a mammalian *in vitro* muscle system in which myofibers mature to this extent [37].

### Translation and trafficking

The MT network with its associated motor proteins are importantly involved in controlling translation and trafficking during muscle cell development and protein turnover of striated muscle cells via spatiotemporal control of the translational machinery; the MT lattice forms tracks upon which mRNA and proteins are transported, serving to maintain specialised subcellular domains of muscle cells, such as t-tubules or the ID (in cardiac muscle) [48, 49]. As such, defective RNA metabolism and protein trafficking underlies a variety of striated muscle diseases [76] Recent studies demonstrate that in both cardiomyocytes and skeletal muscle fibres, mRNA mobility occurs predominantly via MTs, and is dependent on the recruitment of kinesin-1 as diffusion is highly restricted due to the density of sarcomeres in myocytes [76, 77]. mRNAs become trapped in the perinuclear region upon MT depolymerisation, leading to the accumulation of large RNP granules and nascent proteins at the nucleus, causing nuclear export defects and transcriptional inhibition, potentially detrimental to muscle function [76, 77]. Nesprin-1, through AKAP6 mediated MTOC anchorage and recruitment of MT motor protein kinesin-1, is uniquely posed to facilitate association of the MT network in close proximity to exported RNAs and newly synthesised proteins for transport from the nucleus [49]. Nesprin-1 mutations could potentially disrupt perinuclear MT network associations or may impede engagement of kinesin-1 and loading of cargo, to adversely affect the dynamic process of protein trafficking with pathological implications. For example, most NE-related DCM and EDMD patients have conduction defects, often attributed to altered expression and/or overt mislocalisation of connexin 43 (Cx43), a gap junction protein that mediates electrical transmission at the ID of cardiomyocytes [78, 79]. The remodelling of Cx43 is associated with impairment of the MT network, associated with abnormal electrical communication between cardiomyocytes and induced cardiac conduction defects as mimicked in myopathic mouse models. This was demonstrated in the DCM model *Lmna*^H222P/H222P^ mice that showed aberrant remodelling of Cx43 leading to electrical conduction disturbance. In a *Drosophila* model, the perinuclear rim was shown to be the trafficking origin for Cx43 targeting to the ID, through a pathway dependent on MTs via MT-associated proteins: plus-end-tracking protein (+TIP) EB1 and kinesin protein Kif5b. These proteins facilitate Cx43 delivery by tethering MT plus ends at the adherens junctions via N-cadherin and β-catenin. MSP300 (*Drosophila* nesprin-1 orthologue) binds to EB1, and nesprin-1 and -2 interact with KLC1/2 that bind to Kif5b forming a kinesin-1 heterotetramer, illustrating a potential pathway that relates nesprin and the LINC complex to Cx43 trafficking [80–82]. The use of paclitaxel stabilised the MT network and improved conduction defects demonstrating a novel pathophysiological mechanism based on the MT network and Cx43 displacement with a promising therapeutic strategy [78]. Thus, the role of nesprin-1/1α2 and -2 in MT mediated transport and trafficking warrants further investigation as a novel mechanism underlying pathological features of NE myopathies.

### Force transmission

The physical coupling of the cytoskeleton and contractile apparatus to the nucleus by the LINC complex is central to the transmission of forces, and especially relevant to mechanical tissues such as striated muscle, that must generate, withstand, and dynamically adapt to contractile force. Nuclear homeostasis exists as a function of opposing forces conveyed by the different cytoskeletal networks — a push-pull balance of compressive forces mediated by actin and IFs opposed by a resistive MT network that forms a protective perinuclear cage (Figure 3B) [49, 83]. The resistance to compression or an ‘outward force’ is evident as pharmacological disruption of the MTs results in a large expansion of the nuclei *in vitro* [84]*.* Knockdown of desmin or its binding partner nesprin-3 in rat ventricular myocytes results in MT-dependent nuclear involutions that cause DNA damage, loss of association between the nuclear lamina and chromatin, and ultimately large transcriptomic changes. Nuclear infolding was largely prevented by co-expression of dominant negative-KASH which instead caused nuclear expansion, suggesting a model where MTs interacting with nesprin-1 and/or -2 drive nuclear infolding, normally resisted by desmin and nesprin-3 [83]. The role of nesprin in the MT cage was further evidenced in *Drosophila*, as the nesprin-1 orthologue MSP300 conveys elastic properties to the associated structurally rigid nuclear MTs stabilised by Spectraplakin and EB1, which form a flexible perinuclear shield that can withstand contractile forces to maintain nuclear morphology [82]. Disruption to this myonuclear scaffold resulted in aberrant nuclear morphology, affecting nuclear distribution of lamin A/C, lamin B, and heterochromatin protein 1 (HP1). Knockdown of MSP300 in larval body wall muscles of *Drosophila* resulted in loss of myonuclear MT caging and consequently impaired nucleo-cytoskeletal force transmission, as suggested by reduced nuclear displacement in response to force [85]. In isolated nuclei, mechanical stress applied through nesprin-1 resulted in nuclear stiffening, evidencing that force transmission through nesprin-1 can directly influence mechanical properties of the nucleus [86]. *In vitro* biophysical assays in mouse embryonic fibroblasts show that under physiological deformations that mimic mechanically active tissues such as muscle, displacement of nesprin-1 KASH isoforms or nesprin-2α impaired propagation of intracellular forces, causing reduced nuclear deformation in response to extracellular load and disturbed organisation of F-actin and IFs at perinuclear regions [19]. However, it is interesting to note the nesprin-1 CH KO mouse model i.e. lacking the actin binding domains of nesprin-1G, survive normally with no overt striated muscle defects, suggesting nesprin-1G and other CH domain-containing nesprin-1 isoforms are dispensable for muscle structure and function in adult mice [62]. In fact, the dispensability of an intact LINC complex has been recently utilised as a novel therapeutic strategy for multiple models of laminopathy, as impeding force transmission may reduce physical stress on susceptible mutant nuclei with a compromised lamina [60, 87, 88]. In *Lmna* mutant mice, disruption of SUN1, known to preferentially interacts with the MT network, diminished nesprin-1 association at the NE and protected mutant cardiomyocytes from contraction-induced stress, indicating a reduction in force transmission through cardiomyocyte MT network [88]. Likewise, in isolated *Lmna* myofibers, LINC complex disruption by expression of dominant-negative KASH reduced nuclear damage and improved cell viability and contractility; a similar mitigation of damage was seen with MT stabilisation evidencing the relationship of nesprin-1 in transmitting MT-mediated mechanical tension to the nucleus [89].

### Mechanotransduction and signalling pathways

Force transmission to the nucleus via the LINC complex mediates several signalling pathways by modulation of anchored chromatin and mechanosensitive transcription factors, thus mediating a downstream biological response; the process by which mechanical events are converted into biomechanical signals to produce a cellular response is known as mechanotransduction [90]. Nesprin-1 mutations may interrupt physical interactions at the NE as discussed and influence myocyte function via mechanosignalling through structural alterations, such as abnormal nuclear morphology potentially impacting genome organisation, or impaired nuclear integrity and fragility rendering load bearing myonuclei susceptible to mechanical injury and nuclear damage [22, 91, 92]. Cardiomyocytes isolated from nesprin-1 KO mice display misshapen nuclei and NE invaginations, associated with altered heterochromatin density [93]. Separately, cardiomyocytes derived from nesprin-1 or -2 KO mice showed improper response of mechanosensitive genes such *as iex-1, egr-1, c-jun, c-fos and c-myc* under biochemical stimulation, which in turn can modulate cardiac hypertrophy, further suggesting that a physical uncoupling of the LINC complex leads to impaired mechanotransduction in muscle cells [69]. Altered mechanosignalling may also be a consequence of the integration of nesprins within the LINC complex with the nuclear lamina and chromatin, whereby LINC complex-associated components, such as Lamin A/C and emerin, can interact directly with histones or through chromatin associated proteins such as heterochromatin protein 1 (HP1), to regulate genome arrangement and transcriptional regulation [18,19,94–97]. LINC complex disruption may affect the epigenetic status of myocyte nuclei and its transcriptomic profile in response to normal contractile activity or mechanical stress, promoting maladaptive remodelling leading to myocyte dysfunction, or death; the identification of aberrant signalling pathways underlying muscle function may provide targets for therapeutic modulation [98, 99]. Mitogen-activated protein kinase (MAPK) signalling component ERK1/2 is often hyperactivated in NE myopathies and observed in mutants and/or KOs of the genes encoding lamin A/C, emerin, or nesprin-1 [22, 100–102]. In cardiomyocytes, ERK1/2 elevation is associated with aberrant actin organisation and nuclear mispositioning through phosphorylation of FHOD1/3, a recently identified binding partner of giant nesprin-1 and -2 (Table 1) [103–105]. Inhibition of MAPK pathways in several laminopathy mouse models showed improved heart function, and survival indicating the pathological significance of dysregulated MAPK signalling and its value as a therapeutic target [106, 107].

The LINC complex confers mechanical regulation of NPC import and export, via force transmission through the actin cytoskeleton, facilitating an additional hub of mechanosensation at the NE [102]. Stretch-activation of NPCs modulates nuclear/cytosolic shuttling of mechanosensitive transcription factors YAP/TAZ and myocardin-related transcription factor-A (MRTF), which regulate cardiac development and function. Disruption to the LINC complex prevented cytoskeletal coupling to the NPCs thus modulation and translocation of YAP/TAZ and YAP mediated cell proliferation upon strain [108–110]. Nuclear accumulation of YAP/TAZ increased in *Lmna* and nesprin-1-mutant skeletal muscle cells, implicating dysregulation of YAP as a pathogenic contributor in muscle dystrophies [111].

Growing evidence suggests a role for nesprin-1 and -2 and the LINC complex in nuclear Ca^2+^ handling, as abnormal calcium homeostasis is an established pathological feature of muscular dystrophies. In human keratinocytes, nesprin-2 was shown to have a role in Ca^2+^/calmodulin mediated nuclear trafficking, and displacing nesprin-2 from the NE results in elevated cytoplasmic Ca^2+^ levels, caused by a mutation (T6211M) in the C-terminus of nesprin-2, identified in familial EDMD-conduction defects [112]. This region is highly homologous to nesprin-1α2, which as discussed is capable of recruiting AKAP6, a potential molecular candidate for Ca^2+^ regulation at the nucleus [34]. AKAP6 can regulate cardiac hypertrophy pathways by anchorage of cAMP-dependent protein kinase A (PKA), providing spatial and temporal control of cAMP signalling and PKA phosphorylation [113]. AKAP6 also exerts a regulatory effect by anchoring a ‘calcineurin signalosome’ at the NE, engaging downstream factors MEF2D and NFATc, such that C2C12 skeletal myoblast differentiation and neonatal rat ventricular myocyte hypertrophy are inhibited upon targeting of this complex [114–116]. Additionally, the association of nesprin-1α2 and AKAP6 was shown to be necessary for the NE localisation of RyR and AKAP6-mediated cardiac hypertrophy in rat hearts occurred via RyR2 phosphorylation [34]. Increased Ca^2+^ leak via RyRs is another well-established feature of heart failure and laminopathies, demonstrated to be mechanically regulated by MT dependent transmission of stress from the sarcolemma to sarcoplasmic reticulum, which may similarly occur at the nucleus [92, 117]. Nesprin-1α2 and AKAP6 thus potentially form a molecular complex at the NE able to regulate perinuclear Ca^2+^ homeostasis and cardiac remodelling pathways, a potential target of therapeutic modulation.

## Conclusions

Over the past decades, our understanding of the dynamic organisation of the NE has rapidly expanded, revealing that it not only encapsulates and protects the genomic material but forms a hub of mechanosensation, capable of modulating nuclear morphology, genome organisation, gene transcription and signalling pathways. It has been well established that the cooperation of cytoskeletal networks is the cornerstone of mechanotransduction and force transmission in both cardiac and skeletal muscle. Mechanotransduction at the NE is fundamentally mediated by the connection of the cytoskeleton to the nucleus by the LINC complex, central to which are an array of nesprin isoforms and nesprin-binding partners, as summarised in Table 1. In parallel, several pathogenic mutations have been identified in NE components, including several nesprin-1 and -2 mutants manifesting as striated muscle diseases despite their ubiquitous expression.

In recent years, the muscle specific isoform, nesprin-1α2 has emerged as a key regulator of striated muscle myonuclei mechanics. In addition to canonical functions of the LINC complex, nesprin-1α2 performs novel roles at the NE in cardiac and skeletal muscle through interactions with KLC-1/2 and AKAP6 that mediate the perinuclear MT network. Interference to this organisation may have detrimental effects on myogenesis, nuclear positioning and homeostasis, protein trafficking and calcium handling at the nucleus, all of which are commonly maladapted and dysregulated in cardiac and skeletal myopathies. The unique role of nesprin-1α2 evidences the need for thorough investigation into the specificities of nesprin isoforms, so far confounded by a lack of isoform specific antibodies stemming from the high homology between variants. In this review, we have primarily discussed the role of nesprin-1 isoforms and where established, specifically nesprin-1α2, although whether previously recognised roles of nesprins within the LINC components may be attributed to a particular isoform or functional domain remains to be elucidated and so has been included where relevant. Multiple mouse models have clearly demonstrated compensatory abilities and overlapping functions of the multiple isoforms resulting in degrees of dispensability of in adult mice. It is evident that the full scope of roles and redundancies of the numerous nesprin isoforms are yet to be fully discovered; understanding of the nesprin family functions and their extensive interacting protein network at the NE would further provide to the precise cytoskeletal connections to the nucleus and interplay between the sarcomere and the non-sarcomeric cytoskeleton, necessary to decipher the pathophysiological mechanisms underlying nesprin-related muscle diseases.

## Perspectives

- Cooperation of cytoskeletal networks is the cornerstone of mechanotransduction and force transmission in both cardiac and skeletal muscle, mediated by the connection of the cytoskeleton to the nucleus by the LINC complex, central to which are an array of nesprin isoforms and nesprin-binding partners, which mutations are associated with dilated cardiomyopathy and Emery–Dreifuss muscular dystrophy,
- This review is focusing on current knowledge of the novel role of nesprin-1, in particular, muscle specific isoform nesprin-1α2, which has emerged as a key regulator of striated muscle myonuclei positioning and mechanics through its interactions with microtubules in addition to the previously described functions of the LINC complex,
- Future direction will be elucidating the precise cytoskeletal connections to the nucleus via complete understanding of the multiple nesprin isoforms and extensive interacting proteins at the NE and sarcomere, to decipher their numerous roles in striated muscle and pathophysiological mechanisms underlying the nesprin and the LINC-related muscle diseases, informing potential targets of therapeutic modulation.

## Abbreviations

AD: adaptive domain
AKAP: alpha kinase anchoring protein
BAF: barrier-to-autointegration factor
BIN1: amphiphysin 2
CH: calponin homology
EB: end binding
FHOD: formin homology domain-containing proteins
ID: intercalated disc
INM: inner nuclear membrane
KASH: Klarsicht/Anc-1/Syne Homology
KHC: kinesin heavy chain
KLC: kinesin light chain
LINC: linker of nucleoskeleton and cytoskeleton
LRMP: lymphoid restricted membrane protein
MTOC: microtubule organising centre
NFAT: nuclear factor of activated T-cells
PCM: pericentriolar material-1
PCNT: pericentrin
PKA: protein kinase A
PNS: perinuclear space
RNP: ribonucleoprotein
SR: spectrin repeat
SUN: Sad1P/Unc-84
SYNE: synaptic nuclear envelope
+TIP: plus tip-interacting proteins
TA: transcriptional coactivator with A PDZ-binding domain
YAP: yes-associated protein
